# Conjunctival transcriptome profiling of Solomon Islanders with active trachoma in the absence of *Chlamydia trachomatis* infection

**DOI:** 10.1186/s13071-018-2682-2

**Published:** 2018-02-21

**Authors:** Hristina Vasileva, Robert Butcher, Harry Pickering, Oliver Sokana, Kelvin Jack, Anthony W. Solomon, Martin J. Holland, Chrissy h. Roberts

**Affiliations:** 1 Clinical Research Department, London School of Hygiene & Tropical Medicine, Keppel Street, London, WC1E 7HT UK; 2 Eye Department, Solomon Islands Ministry of Health and Medical Services, PO Box 349, Honiara, Solomon Islands

**Keywords:** Solomon Islands, Trachoma, Transcriptome, *Chlamydia trachomatis*

## Abstract

**Background:**

Clinical signs of active (inflammatory) trachoma are found in many children in the Solomon Islands, but the majority of these individuals have no serological evidence of previous infection with *Chlamydia trachomatis*. In Temotu and Rennell and Bellona provinces, ocular infections with *C. trachomatis* were seldom detected among children with active trachoma; a similar lack of association was seen between active trachoma and other common bacterial and viral causes of follicular conjunctivitis. Here, we set out to characterise patterns of gene expression at the conjunctivae of children in these provinces with and without clinical signs of trachomatous inflammation-follicular (TF) and *C. trachomatis* infection.

**Methods:**

Purified RNA from children with and without active trachoma was run on Affymetrix GeneChip Human Transcriptome Array 2.0 microarrays. Profiles were compared between individuals with ocular *C. trachomatis* infection and TF (group DI; *n* = 6), individuals with TF but no *C. trachomatis* infection (group D; *n* = 7), and individuals without TF or *C. trachomatis* infection (group N; *n* = 7). Differential gene expression and gene set enrichment for pathway membership were assessed.

**Results:**

Conjunctival gene expression profiles were more similar within-group than between-group. Principal components analysis indicated that the first and second principal components combined explained almost 50% of the variance in the dataset. When comparing the DI group to the N group, genes involved in T-cell proliferation, B-cell signalling and CD8+ T cell signalling pathways were differentially regulated. When comparing the DI group to the D group, CD8+ T-cell regulation, interferon-gamma and IL17 production pathways were enriched. Genes involved in RNA transcription and translation pathways were upregulated when comparing the D group to the N group.

**Conclusions:**

Gene expression profiles in children in the Solomon Islands indicate immune responses consistent with bacterial infection when TF and *C. trachomatis* infection are concurrent. The transcriptomes of children with TF but without identified infection were not consistent with allergic or viral conjunctivitis.

## Background

Trachoma, caused by *Chlamydia trachomatis* (*Ct*), is the most common infectious cause of blindness worldwide, responsible for an estimated 1.9 million cases of blindness or visual impairment [1]. Ocular infection with *Ct* predominantly occurs in young children and triggers follicular and papillary inflammation. Repeated rounds of infection, inflammation and disease resolution lead to deposition of scar tissue on the conjunctiva (trachomatous scarring, TS) which accumulates with time; ultimately distorting the eyelid and, in severe cases, bringing the eyelashes into contact with the globe of the eye (trachomatous trichiasis, TT). Abrasion by these lashes can cause opacity (corneal opacity, CO) and blindness. Trachoma is treated with a package of interventions aimed at controlling infection and reducing the risk of blindness from TT, collectively termed the SAFE strategy. These include eyelid Surgery for those with TT, community-wide Antibiotic distribution, promotion of Facial cleanliness and Environmental improvement [2].

In a 2013 survey of children aged 1–9 years in Temotu and Rennell and Bellona provinces of the Solomon Islands, we found that more than a quarter (26.1%) of those examined had the active (inflammatory) trachoma sign trachomatous inflammation–follicular (TF). This level of endemicity was high enough to warrant treatment of the whole population by mass drug (azithromycin) administration, but the prevalence of the more severe active trachoma sign trachomatous inflammation–intense (TI; 0.2%) and ocular *Ct* infection (1.3%) was unusually low given the TF prevalence [3]. In two consecutive surveys of this population we consistently found that over 90% of TF cases occurred in individuals who had no PCR detectable ocular *Ct* infection. We also assessed the blood levels of anti-Pgp3 antibodies, a putative serological marker of lifetime *Ct* exposure [4]. We found that TF was not associated with Pgp3 seropositivity. These data suggested that the majority of TF cases that we identified were in individuals who were very unlikely to have ever been exposed to any form of *Ct* infection [5]. When we tested for the presence of several other infectious micro-organisms that are known to be able to cause symptoms of follicular inflammation (*Haemophilus influenzae*, *Streptococcus pneumoniae*, *Moraxella catarrhalis*, *Staphylococcus* spp. and *Adenoviridae*), we found no association between TF and any of these bacteria and viruses. A broad screen for changes in bacterial communities of the conjunctiva in TF cases was similarly null [6]. It could therefore be reasonably concluded that TF in this population is unlikely to be linked to any bacterial infection.

The grading of TF was conducted by graders trained to the same international standards as graders in other countries, giving us no reason to suspect the TF phenotype in the Solomon Islands is different to that in trachoma-endemic populations elsewhere in the world [3]. However, we observed very little TS in the Solomon Islands [5]. Therefore, an important question that emerges from our work is whether there are differences in the underlying immune response that could explain why, in the Solomon Islands, highly prevalent TF does not seem to be concurrent with the same burden of blinding sequelae of trachoma as in other countries.

Previous work has described the typical host immune response to ocular infection with *Ct,* a response that can persist for weeks to months after the infection is resolved [7]. While the frequency and duration of *Ct* infection decrease with age, inflammation can be found in a significant fraction of older people, and is associated with progression of scarring [7, 8]. The clearance of Ct infection is generally accepted to be mediated by interferon gamma (IFNγ) [9–11] with epithelial and lymphoid cells generating a strong pro-inflammatory Type 1 response that includes production of growth factors, such as platelet-derived growth factor (PDGF), connective-tissue growth factor (CTGF) and tumour necrosis factor alpha (TNFα) [12–15]. Prolonged activation of these responses leads to the formation of the lymphoid follicles that characterise TF. Studies have also shown upregulation of major histocompatibility complex (MHC) class I expression, the induction of MHC class II in cells in which it is normally absent, as well as the expression of genes typical of neutrophils and natural killer (NK) cell cytotoxicity [13, 16]. Other types of conjunctivitis (i.e. those not caused by *Ct* infection) have different underlying immune pathologies and are characterised by quite distinct transcriptomic signatures. Allergic conjunctivitis, for instance, is characterised by eosinophilic inflammation, mast cell degranulation, upregulation of adhesion molecules and production of chemokines [17–19]. Conversely, in vitro transcriptional profiling studies of adenoviral infection of human epithelial cells indicate dominance of anti-viral and type-one interferon-associated pathways [20].

We hypothesised that gene expression profiles of TF in the Solomon Islands could help us to determine whether TF in children from the Solomon Islands is caused by bacteria, viruses or allergens.

## Methods

### Specimen collection

Specimens were collected during a population-based prevalence survey for trachoma in Temotu and Rennell and Bellona provinces of the Solomon Islands which took place in 2013 [3]. Clinical grades were assigned in the field by Global Trachoma Mapping Project-certified graders according to the WHO simplified grading scheme [21, 22]. All clinical data and methods relating to the 2013 survey have been published elsewhere [3]. Briefly, we used polyester-coated cotton swabs to collect conjunctival specimens from 1002 1–9-year-olds. Swabs were collected from the everted right conjunctiva and placed immediately into 300 μl RNAlater, then kept cool in the field and frozen within 48 h of collection. Specimens were shipped on dry ice to the UK for processing.

### Case-control selection

From the whole population sample, three subsets of specimens were selected for the microarray-based gene expression profiling experiment. Group N (*n* = 7) were children who had neither TF, nor *Ct* infection [3]*,* nor any of other common ocular infections (as listed in the introduction) [6]. Group D (*n* = 7) were children who did have TF, but had neither *Ct* nor any of the other infections.

Two years after the initial survey, all individuals in groups D and N were revisited by chance during a serological survey and were tested for evidence of prior *Ct* infections with an anti-Pgp3 ELISA test [5]. All members of groups D and N were seronegative at that time, suggesting that no member of either group had previously been infected with *Ct*. The third group DI (*n* = 6) had both clinical signs of TF (but not TI) and current ocular *Ct* infection during the 2013 survey. The mean *Ct* load in the 6 DI conjunctival samples was 338 *omcB* copies/μl (range: 0.4–1121 *omcB* copies/μl). The detected strain in all 6 DI samples were serovar C according to *ompA* sequence, and were most closely related to *Ct* A/HAR-13 within the T2 ocular clade when aligned at the whole-genome level [3]. We had very few *Ct* infection cases to choose from, so we were unable to stringently filter the DI group to ensure that no ‘other infections’ were present. Three of the DI group members had no other infections besides *Ct*, one had *S. pneumoniae* present, one had *H. influenzae*, and one had *Adenovirus* and *H. influenzae* present. All of these infections were low load (< 5 copies/μl). None of the six members of group DI participated in the 2015 follow-up survey. Participants were age- and gender-matched between groups (Kruskall-Wallis test, *χ*^2^ = 0.31804, *df* = 2, *P* = 0.853 and *χ*^2^ = 2.6412, *df* = 2, *P* = 0.267, respectively).

### RNA extraction and quantification

DNA and total RNA from each sample were simultaneously extracted using the Qiagen AllPrep Mini protocol (Qiagen, Hilden, Germany). DNA extracts were tested for *Ct* infection using a droplet digital PCR (ddPCR) assay [23] which we have previously used in both high [24, 25] and low endemicity settings [3, 26]. The diagnostic performance of the in-house ddPCR assay, published elsewhere, has a demonstrated sensitivity of 97.1% and specificity of 90.0% in low endemicity areas [27]. Purified RNA was stored at -80 °C before testing. The quantity and quality of RNA was calculated using Agilent RNA 6000 Pico Assay according to the manufacturer’s protocol (Agilent, Santa Clara, USA).

### Probe library preparation

RNA samples were normalised and used as a template for the generation of cDNA using NuGEN Ovation Pico WTA System V2 protocol (NuGEN, Leek, Netherlands). cDNA was amplified and purified using Agencourt RNAClean XP (Beckman Coulter, High Wycombe, UK) followed by QIAGEN QIAquick PCR Purification (Qiagen, Hilden, Germany).

cDNA fragmentation and labelling was performed according to FL-Ovation™ cDNA Biotin Module V2 protocol (NuGEN, Leek, Netherlands). Biotinylated cDNA was hybridised to GeneChip HTA 2.0 microarrays according to the NuGEN Hybridization, Cocktail Assembly and Fluidics Protocol for single arrays (NuGEN, Leek, Netherlands). The hybridized arrays were washed and stained with streptavidin-phycoerythrin (SAPE) in a Fluidex GeneChip 450 according to the GeneChip® Expression Analysis protocol (Thermo Fisher Scientific, Hemel Hempsted, UK). Goat anti-biotin-SAPE antibodies (IgG) were used for signal amplification.

### Microarray data processing

The GeneChip HTA 2.0 array contains approximately 1.7 million probe sets, represented by 70,523 human transcript clusters [28]. Analysis of array data was conducted using Bioconductor and R software packages [29, 30]. Mean microarray average plots were visually inspected to ensure consistency and quality of hybridization intensity between arrays. Array data files were normalized using Robust Multichip Average (RMA) algorithms from the Bioconductor package “oligo” [31]. Transcript clusters were annotated using the ‘hta20transcriptcluster.db’ Bioconductor package. Normalized transcript clusters without annotation information, those with no detected signal and array endogenous controls were discarded from the overall data set. Where more than one transcript cluster mapped to a single accession number, the transcript cluster with the highest fluorescence intensity was retained and the others discarded. Of the resultant transcript clusters, those with an RMA-normalised intensity interquartile range of < 0.1 across all samples were also removed [30].

### Identification of differentially expressed genes

Group mean hybridization intensities of each transcript were compared in a pairwise manner between the DI, D and N sample groups, applying empirical Bayes moderated t-test for mean difference [32] using the Bioconductor package “limma” [33]. To account for multiple testing of large number of variables, a corrected *P*-value for each transcript was obtained by subjecting the *P*-values from each comparison to a permutation analysis [34]. Transcript clusters with a corrected *P*-value lower than the selected threshold of < 0.05 for each comparison were considered differentially expressed (DE) [35]. Directionality of DE gene regulation was determined by gene log2 fold change (log2FC) of expression fluctuating above (upregulated) and below (downregulated) log2FC = 1. DE genes from all three comparisons (DI vs D, DI vs N, D vs N) were combined for the purposes of visualizing the differences in global gene expression between groups. Principal components analysis (PCA) was used to visualise differences in transcriptomes between samples according to variance between gene expression levels of total DE genes [36]. PCA was used to identify whether the phenotypic groups could be separated according to variance in total DE genes expression profiles. Group separation was tested using ordinal logistic regression, using the “MASS” package in R [37].

### Biological function of differentially expressed genes

Lists of DE gene GenBank accession numbers were subjected to Gene Ontology (GO) terms analysis, using Database for Annotation, Visualization and Integrated Discovery (DAVID) v6.8 [38]. GO terms were considered statistically significant based on Benjamini’s adjusted *P*-value < 0.05. The top five statistically significant genetic pathways for the DE genes in each comparison were identified using Kyoto Encyclopaedia of Genes and Genomes (KEGG) bioinformatics database [39].

## Results and Discussion

**Fig. 1:**
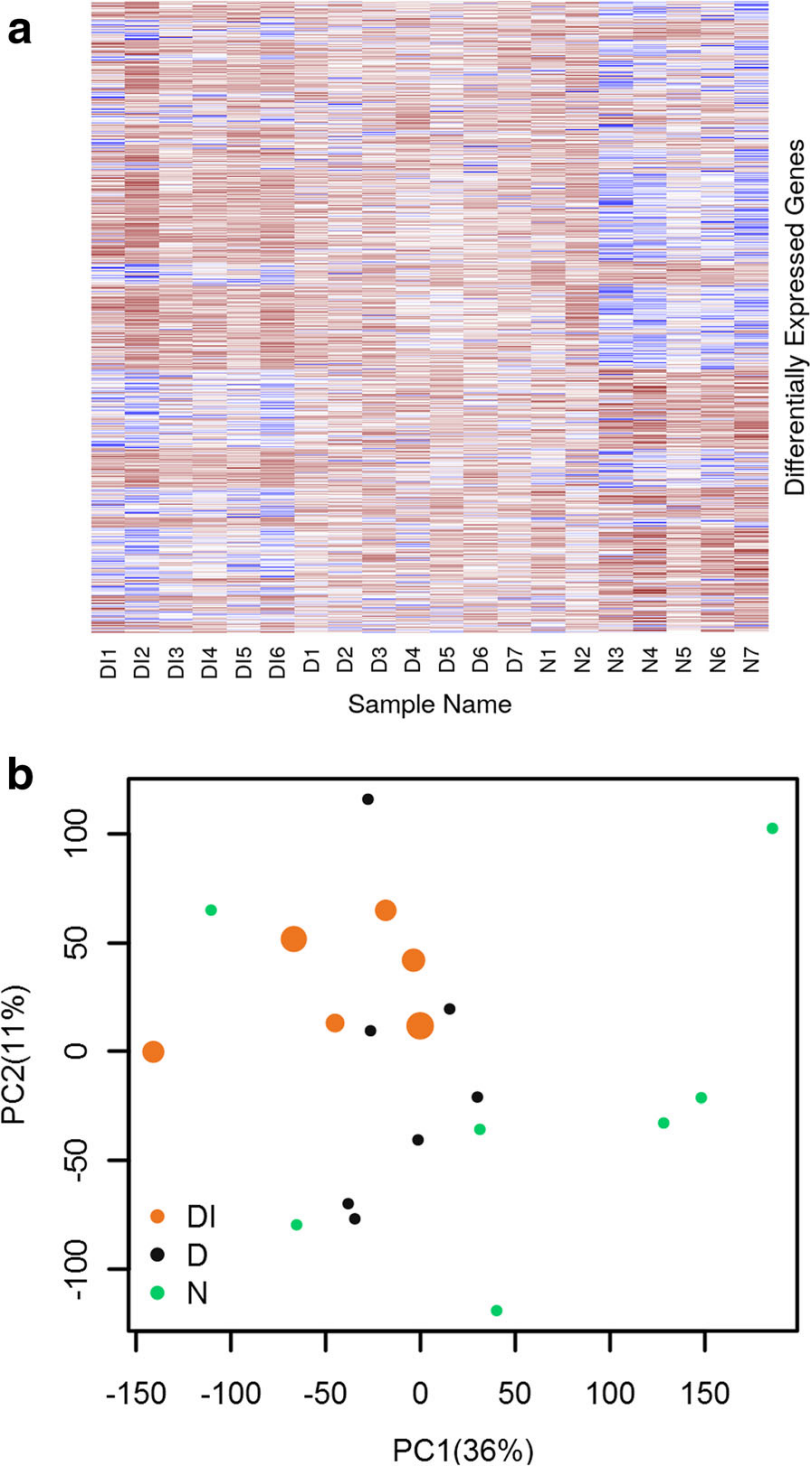
**a** Heat map visually representing the gene expression intensity of total differentially expressed genes, (*n* = 7761) in each array sample. Red indicates high expression, blue indicates low expression. **b** Principal components analysis of differentially expressed genes in children with and without TF and *Ct* infection in the Solomon Islands (*n* = 20). Point colour indicates the clinical phenotype whilst the size of the point is proportional to the load of *Ct* infection in specimens within the DI group

Following comparison of transcript clusters hybridisation intensities between groups, a combined total of 7761 DE genes were identified. The number of genes per group is summarized in Table 1 and the comparison of DE gene expression across all samples is visualised as a heat map in Fig. 1a. Most samples appeared to be more similar within-group than between-groups, with some exceptions (samples DI3, DI4, N1 and N2). There were no significant differences between chlamydial load (Mann-Whitney U-test, *U* = 3, *n*_*1*_ = 2, *n*_*2*_ = 4, *P* = 0.8), nor between the age or gender of the participants from whom samples DI3 and DI4 came from, compared with the rest of the DI group specimens. The same was observed for samples N1 and N2 compared with the other samples from the N group. As no significant differences in the demographics of the group members that might account for the observed differences in gene expression pattern can be identified, we suggest this is likely to be due to natural within-group heterogeneity. Figure 1b shows a bi-plot of the first two principal components (PCs) which cumulatively describe 47% (PC1: 36%, PC2: 11%) of the total between-group variance in gene expression. Each coloured point represents one of the specimens. There are visible separations between clusters of specimens which have similar phenotypes.

**Table 1:** Differentially expressed (DE) genes in each group comparison, separated according to the direction and magnitude of change

	Comparison	DI vs D (*n* = 2368)	DI vs N (*n* = 6371)	D vs N (*n* = 1933)
Upregulated genes	*n* (%)	1388 (58.6)	2777 (43.6)	700 (36.2)
	log2FC, mean (min-max)	0.378 (0.067–2.971)	0.437 (0.072–2.694)	0.322 (0.064–1.477)
Downregulated genes	*n* (%)	980 (41.4)	3594 (56.4)	1233 (63.8)
	log2FC, mean (min-max)	−0.346 (− 3.686– −0.065)	−0.294 (− 3.901– −0.054)	−0.230 (−1.436– −0.054)

We observed substantial gene expression profile differences between the DI, D and N groups (Fig. 1). The KEGG pathway analysis is summarised in Table 2. Our data show that the most enriched pathways observed when comparing the DI group with the D and N groups are closely linked to key elements of the immune response, including IL17 and IFNγ production, genes controlling T-cell proliferation/response and B-cell signalling. The D group, which had no evidence of current or previous *Ct* infection, was not enriched for the same pathways when compared to the N group. Surprisingly, individuals with visible sub-conjunctival lymphoid follicles had no enrichment of pathways related to lymphocyte activation, nor upregulated genes associated with activation and recruitment of NK and dendritic cells (*CCL18*), fundamental for TF [13]. In the *Ct*-infected (DI) group we saw some patterns of pathway enrichment that closely resembled data from other trachoma-endemic populations [8, 13]. However, whilst IL17 and IFNγ pathways were upregulated in the Solomon Islands, key pro-fibrotic markers and genes encoding extracellular matrix molecules (*MMP7, MMP9, MMP12*) were not found to be differentially regulated, nor were their associated pathways enriched [13]. These pathways are typical of scarring disease, but are also upregulated during and after *Ct* infection in young people with inflammatory trachoma [40].

**Table 2:** Biological pathways (GO terms and KEGG pathways) showing significant enrichment across three between-group comparisons

Comparison (No. of genes)	Principal GO terms	Fold enrichment	Adjusted *P*-value	Principal KEGG pathways	*P*-value
DI vs D (*n* = 2368)	GO:0039692: ssRNA viral replication	6.93	2.23 × 10^−2^	hsa04658: Th1 and Th2 cell differentiation	6.80 × 10^−7^
	GO:2,001,185: regulation of CD8+ T-cells	6.93	2.23 × 10^−2^	hsa04110: Cell cycle	4.33 × 10^−6^
	GO:0072643: IFNγ secretion	5.78	2.34 × 10^−3^	hsa05203: Viral carcinogenesis	1.40 × 10^−4^
	GO:0007076: mitotic chromosome condensation	5.39	2.70 × 10^−2^	hsa04114: Oocyte meiosis	2.19 × 10^−4^
	GO:0046633: T-cell proliferation	5.33	2.65 × 10^−2^	hsa04612: Antigen processing and presentation	3.16 × 10^−4^
	GO:0032620: interleukin-17 production	4.16	2.30 × 10^−2^	hsa05166: HTLV-I infection	1.33 × 10^−3^
	GO:1,901,976: regulation of cell cycle	3.50	3.65 × 10^−2^	hsa05340: Primary immunodeficiency	3.12 × 10^−03^
DI vs N (*n* = 6371)	GO:0036037: CD8+ T-cell activation	2.88	3.59 × 10^−3^	hsa04750: Inflammatory mediator regulation of TRP channels	1.22 × 10^−3^
	GO:0006270: DNA replication initiation	2.41	4.18 × 10^−3^	hsa03030: DNA replication	2.06 × 10^−3^
	GO:0045047: Protein targeting to ER	2.07	1.11 × 10^−5^	hsa03040: Spliceosome	2.06 × 10^−3^
	GO:0019080: Viral gene expression	1.98	4.53 × 10^−9^	hsa04612: Antigen processing and presentation	2.38 × 10^−3^
	GO:0050853: B-cell receptor signalling pathway	1.83	3.19 × 10^−2^	hsa04925: Aldosteron synthesis and secretion	3.02 × 10^−3^
	GO:0048477: oogenesis	1.74	2.60 × 10^−2^	hsa04658: Th1 and Th2 cell differentiation	5.50 × 10^−3^
	GO:0042098: T-cell proliferation	1.65	6.17 × 10^−4^	hsa04110: Cell cycle	5.70 × 10^−3^
D vs N (*n* = 1933)	GO:0045047: Protein targeting to ER	6.59	2.41 × 10^−20^	hsa03010: Ribosome	1.18 × 10^−11^
	GO:0019083: Viral gene expression	4.37	1.89 × 10^−18^	hsa04612: Antigen processing and presentation	1.34 × 10^−3^
	GO:0042255: ribosome assembly	3.92	9.50 × 10^−4^	hsa03030: DNA replication	2.42 × 10^−2^
	GO:0042254: ribosome biogenesis	2.50	8.99 × 10^−8^	hsa03040: Spliceosome	5.53 × 10^−2^
	GO:0043043: peptide biosynthetic process	1.92	1.37 × 10^−6^	hsa05332: Graft-versus-host disease	5.53 × 10^−2^
	GO:0006396: RNA processing	1.81	2.26 × 10^−7^	hsa05416: Viral myocarditis	8.33 × 10^−2^
	GO:0033365: Protein localization to organelle	1.63	1.62 × 10^−4^		

Previous studies have shown that thymic stromal lymphoprotein (TSLP), *IL-4, IL-5* and *IL-13* are upregulated during episodes of allergic or seasonal conjunctivitis [41], but these were not significantly differentially regulated in the D group when compared to the N group nor the DI group when compared to the D group. We did not identify any key pathways associated with eosinophilic inflammation, IgE release or degranulation of mast cells and we therefore do not suspect allergic responses to be playing a significant role in the TF phenotype in these children. The predominant pathways that showed highly significant enrichment in the D group, when compared with the N group, were viral gene expression pathways as well as protein biosynthesis pathways centring around ribosome function. However, anti-viral immunity and type one interferon-dependent pathways were not enriched according to this analysis. Viral pathways were also enriched in the DI group when compared to the N and D groups, although these were less significant. There is deep redundancy and overlap of gene content in the GO and KEGG pathways, which means that their names can often be misleading with regards to their roles in a specific clinical context. On that basis, we do not have strong evidence that an as-yet uncharacterised viral infection may be responsible for the observed discrepancy between phenotype and *Ct* infection. This study was limited by small sample size and in some cases the additional diagnosis of other ocular infections that can cause TF-like clinical signs. However, the natural heterogeneity of the data is typical of complex human disease studies and the transcriptional profiles of those with disease and *Ct* infection were sufficiently similar to those seen in other populations.

The World Health Organization guidelines for implementing mass drug administration (MDA) are based largely on TF prevalence. We have previously argued that whilst the Solomon Islands has sufficiently prevalent clinical signs (TF) of trachoma to qualify for implementation of MDA, the prevalence of infection and trichiasis [3, 5], as well as severe scarring and serological signs of prior infection [6] are all too low to suggest that clinical diagnosis with TF has the necessary specificity to be used as an indicator of need for intervention in this population. By showing that the transcriptional profile of TF (*Ct* uninfected) cases in the Solomon Islands share some, but not all, the components of typical trachoma responses seen elsewhere, the current findings add to those of our previous studies to suggest that the majority of TF disease we observe there is not related to *Ct*. We believe that there is potential that similar disease could occur elsewhere and would recommend that a diagnostic test for ocular infection should be considered for routine use in combination with clinical signs of the disease, in order to better inform the decision to treat a population with MDA.

## Conclusions

Our recent studies identified that the majority of TF disease in the Solomon Islands could be attributed to neither *Ct* [3], nor any of several common ocular microbes, nor polymicrobial community [6]. We hypothesised that TF in the absence of current *Ct* infection in the Solomon Island population would have a transcriptional profile that could indicate either an allergic or viral trigger. The host responses we measured did not provide any indication for the involvement of an allergic response, nor was there convincing evidence for a response to a viral infection. The results suggest that further studies in to the aetiology of disease in this context are warranted.

## Abbreviations

CO: Corneal opacity
*Ct*: *Chlamydia trachomatis*
D: Study participant group with TF but no *Ct* infection
ddPCR: Droplet digital polymerase chain reaction
DE: Differentially expressed
DI: Study participant group with concurrent TF and *Ct* infection
GO: Gene Ontology
KEGG: Kyoto Encyclopaedia of Genes and Genomes
Log2FC: Log (base 2) fold change
MDA: Mass drug administration
N: Study participant group with neither TF nor *Ct* infection
PC: Principal Component
PCA: Principal Components Analysis
RMA: Robust multichip average
SAFE: Surgery, Antibiotics, promotion of Facial hygiene and Environmental improvement
TF: Trachomatous inflammation-follicular
TI: Trachomatous inflammation-intense
TS: Trachomatous scarring
TT: Trachomatous trichiasis
WHO: World Health Organization
